# Spermatophyta (Plantae) and invasive alien plants of Wanda Mountains in China: a first checklist

**DOI:** 10.3897/BDJ.11.e104648

**Published:** 2023-06-07

**Authors:** Siqi Wang, Xueshi Wang, Rui Xu, Hongfeng Wang

**Affiliations:** 1 School of Forestry, Northeast Forestry University, Harbin, China School of Forestry, Northeast Forestry University Harbin China; 2 ChengDu Yecool Group Habitat Cultural Communication Co., Ltd, Chengdu, China ChengDu Yecool Group Habitat Cultural Communication Co., Ltd Chengdu China; 3 San Jose State University, San jose, United States of America San Jose State University San jose United States of America

**Keywords:** Wanda Mountains, checklist, invasive, northeast China

## Abstract

**Background:**

China is one of the most biodiverse countries in the world and has given birth to unique ecosystems, abundant species and rich genetic variety. More and more attention has been paid to biodiversity research in China. The Wanda Mountains, located in the east of Heilongjiang Province in northeast Chia, is a northern extension of the Changbai Mountains, one of the main mountains ranges in the region. In this study, we present the first checklist of spermatophyte and invasive alien plant species in the Wanda Mountains, which was compiled using published materials, specimen records and field surveys conducted from 2018 to 2020. This checklist, which has been published in the Global Biodiversity Information Facility (GBIF), provides a comprehensive overview of the plant species richness of the Wanda Mountains.

**New information:**

This data paper presents the first checklist of spermatophytes and invasive alien plants in the Wanda Mountains, comprising a total of 704 species and infraspecific taxa. Amongst these, there are 656 native plants belonging to 328 genera and 94 families and 48 invasive alien plants belonging to 39 genera and 20 families. The checklist includes 251 new records of native plants and 39 new records of invasive plants. This is the first widely shared data on an independent floristic unit in northeast China and can serve as a valuable resource for future biodiversity research in this region and, moreover, trigger more biodiversity data papers in this data-valued country.

## Introduction

As the United Nations develops a post-2020 global biodiversity framework for the Convention on Biological Diversity, attention is focusing on what kind of data could contribute to management remedies for ecosystem conservation to serve its vision of ‘living in harmony with nature’ (Nicholson et al. 2021, Keith et al. 2022). Facing enhanced global ecosystem degradation, biological globalisation and local human pressure, fully understanding data on floristic units, such as a checklist, will be of outstanding value for comprehending and then hopefully managing the rapid change in ecosystem and its biodiversity (Armonies et al. 2018). Given the important role of plants in the ecosystem, a plant checklist provides indispensable basic data that will assist in establishing conservation actions and priorities, providing a support to theory and practice of ecosystem restoration (Shetu et al. 2018). More importantly, checklists can be instrumental in providing tools for additional floristic surveys and biodiversity treatments (Francisco-Ortega et al. 2010).

Although the significance of a checklist is self-evident, there remains a large number of important floristic units lacking basic data obtained from field surveys, such as the Wanda Mountains located in north-eastern China (Fig. 4Wang et al. 2016). With respect to the flora and vegetation of the Wanda Mountains, both are generally considered as a part of the Changbai Mountains and have, therefore, never been treated independently as an individual floristic region of north-eastern China (Zhou et al. 1997, Wang et al. 2016). However, they underwent different processes of formation. Multiple volcanic eruptions have taken place in Changbai Mountains, which has obvious characteristics of primary succession in its plant composition (Fu et al. 1995). In contrast, volcanic influence never reached the Wanda Mountains, so the formation process of its flora has never been interrupted (Wang et al. 2016). In terms of geographical location and climate, the east of Changbai Mountains is close to the Sea of Japan and the south is adjacent to the north China floristic region, which makes it warm and abundant in rainfall (800 - 1800 mm) (Fu et al. 1995, Zhou et al. 1997). In comparison, the Wanda Mountains is located in the north of Changbai Mountains, next to the Xiaoxing'an Mountains, which are colder and drier (500 - 800 mm) (Zhou et al. 1997, Jiang et al. 2005). The difference in altitude is also greater: with the Changbai Mountains having the only alpine tundra region in northeast China above 2100 m and the main peak standing at 2,750 m (Fu et al. 1995, Zhou et al. 1997). In contrast, the average altitude of the Wanda Mountains' hills is only approximately 300 m and the main peak stands at only 854 m (Jiang et al. 2005). Although the Wanda Mountain can be regarded as an independent floristic unit, it has not been given corresponding attention. To date, no flora, checklist or atlas of the Wanda Mountains has been published and no important plant conservation area has been established in this area.

In this study, we compiled the first checklist of spermatophyte and invasive alien plants of Wanda Mountains. Consequently we updated the scientific names and taxonomic treatments by using several bibliographic/taxonomic database resources, including the Catalogue of Life, China (The Biodiversity Committee of Chinese Academy of Sciences 2021), the International Plant Name Index (IPNI 2022), The World Flora Online (WFO 2022), Plants of the World Online (POWO 2022), Flora of China website (FOC, Institute of Botany, the Chinese Academy of Sciences 2019) and APGIV system (The Angiosperm Phylogeny Group 2016). Invasive plants mainly refer to Alien Invasive Flora of China (Ma 2020), The Survey Reports on Chinese Alien Invasive Plants (Ma 2014), various floras, specimens and survey materials. We also marked the plants that have not been recorded as ‘new record’ against local floras, which are ‘Flora Heilongjiangensis’ (Zhou 2003), ‘Ligneous Flora of Heilongjiang’ (Zhou 1986), ‘Flora Plantarum Medicinalium Chinae Boreali-Orientalis’ (Liou 1959), ‘Flora Plantarum Herbacearum Chinae Boreali–Orientalis’ (Liou 2004), ‘Atlas of Northeast Plant Distribution’ (Cao et al. 2019), as well as specimen records from CVH (Chinese Virtual Herbarium) (CVH 2020).

This checklist of Wanda Mountains, obtained through published materials, specimens as well as field surveys, can pave the way for species threat abatement, recovery metrics and more extensive floristic studies and can also provide clues for considering Wanda Mountains as an independent unit in the context of the broader ecosystem recovery.

## General description

### Purpose

This study aims to provide the first checklist of spermatophyte and invasive alien plants of Wanda Mountains (northeast China), which will be updated regularly with new records discovered in this area via GBIF (Wang and Wang 2022). As the Wanda Mountains’ first plant checklist created via published materials, specimen records as well as field survey, these data can deliver reliable basic data for more extensive study of biodiversity, available for use by scientists, researchers or the general public.

## Project description

### Title

Natural Science Foundation of Heilongjiang Province of China C2018004; the project BIFA5_031.

### Personnel

The researchers involved in this project are Siqi Wang (sample-plot survey, taxonomic identification, data collation, essay writing) and Dr. Hongfeng Wang (experimental design, funding, sample-plot survey, taxonomic identification, data analysis) from the School of Forestry, Northeast Forestry University, Harbin, China; Xueshi Wang (Data collation) from the ChengDu Yecool Group Habitat Cultural Communication Co., Ltd, Chengdu, China and Rui Xu (data collation) from San Jose State University, San Jose, United States of America.

### Study area description

Located in the east of Heilongjiang Province, the Wanda Mountains are a northern extension of the Changbai Mountains, which is one of the main mountain ranges in the east of Heilongjiang Province. The administrative region includes six complete cities or counties, including Shuangyashan, Qitaihe, Baoqing, Huanan, Boli and Hulin and a part of six cities or counties, including Jixi, Raohe, Mishan, Jidong, Linkou and Yilan (Wang and Sun 2012). The main vein of Wanda Mountains runs from northeast to southwest, extending from Raoli River in the northwest to Wusuli River in the east and then to Guokui Mountain in the south. It is the watershed between Raoli River and Muling River, about 350 km long from east to west and 250 km wide from north to south (Yang and Yang 2017). The Wanda Mountains are low mountain and hilly areas with an altitude of 300 ~ 500 m and an average gradient of 10° ~ 15° (Guo et al. 2008). Shending Mountain, the main peak of Wanda Mountains, located at the north end of the mountain range, is at an altitude of 831 m. The brown forest, presenting moderate fertility, is the main soil type in the Wanda Mountains, comprising 30 cm to 50 cm thick layer. The Wanda Mountains exhibit an oceanic temperate monsoon climate, with the rain and heat in the same season. The annual average temperature is 2.4°C and precipitation is 500 ~ 800 mm. The coldest month is January, with the monthly average temperature of - 19.4°C. The hottest month is July, with the monthly average temperature of 21.1°C. The frostless period lasts for 125 ~ 150 days and annual accumulated temperature is approximately 2,300 ~ 3,000˚C (Jiang et al. 2005, Wang et al. 2016).

### Design description

This project is designed to obtain the first checklist of spermatophyte and invasive alien plants of Wanda Mountains through published materials, specimen records, as well as field survey.

### Funding

Natural Science Foundation of Heilongjiang Province of China C2018004;

The Strategic Priority Research Program of the Chinese Academy of Sciences, Grant No. XDA19050404;

Cleaning and digitizing plant specimen records from Heilongjiang Province, the project BIFA5_031.

## Sampling methods

### Study extent

From 2018 to 2020, we surveyed the forest-covered region of the Wanda Mountains, with an area over 47,785 km^2^, including 12 administrative districts.

### Sampling description

The data presented in this checklist come from specimen records, local floras and field surveys. We did not find any records in GBIF, but 1767 records in the Chinese Virtual Herbarium. In order to fully understand the plant species diversity in Wanda Mountains, transect lines and quadrats were set up in this area from 2018 to 2020. During the setting of transects and quadrats, we carefully considered the differences of species distribution in different altitudes, precipitation, vegetation, habitats and human disturbance areas and finally determined 23 transects (about 230 km long in total) and 212 quadrat spots to cover all the above areas (Fig. 5). On each spot, we set up one survey quadrat for arborous plants (25 m × 25 m), two for shrub plants (5 m × 5 m) and four for herbaceous plants (1 m × 1 m). In total, there are 1484 quadrats consisting of 212 arborous quadrats, 424 shrub quadrats and 848 herbaceous quadrats. All species, both in quadrats and transect lines, have been recorded. Local floras we used include ‘Flora Heilongjiangensis’ (Zhou 2003), ‘Ligneous Flora of Heilongjiang’ (Zhou 1986), ‘Flora Plantarum Medicinalium Chinae Boreali-Orientalis’ (Liou 1959), ‘Flora Plantarum Herbacearum Chinae Boreali–Orientalis’ (Liou 2004) and ‘Atlas of Northeast Plant Distribution’ (Cao et al. 2019). Invasive plants mainly refer to Alien Invasive Flora of China (Ma 2020), The Survey Reports on Chinese Alien Invasive Plants (Ma 2014), various floras, specimens and survey materials.

### Quality control

1. For all species and infraspecific taxa, photographs were taken and made for specimens being collected in the NEFI (Herbarium of Northeast Forestry University) and IFP (Northeast Biological Herbarium of Shenyang Institute of Applied Ecology, China Academy of Sciences) to provide an important reference for identification.

2.The scientific names in this checklist are based on the identification by an experienced expert in the field of the taxonomy of plants in northeast China, namely, Dr. Hongfeng Wang from the Forestry School of Northeast Forestry University.

3.To obtain a list of species with currently accepted nomenclature, we updated them to match the APGIV classification of angiosperm families (The Angiosperm Phylogeny Group 2016) and all scientific names were checked against online databases (http://tnrs.iplantcollaborative.org/index.html, http://ipni.org, http://plants.jstor.org, https://powo.science.kew.org).

### Step description

1. Obtain the preliminary checklist through published materials, specimen records as well as field survey;

2. Correct the taxonomic status and scientific names to obtain the final checklist;

3. Sort and identify the data, based on published documents such as local floras and reports on invasive alien plants, as well as specimen records.

## Geographic coverage

### Description

The Wanda Mountains

### Coordinates

N 44°51′13″ and N 47°10′30″ Latitude; E 129°30′20″ and E 134°10′10″ Longitude.

## Taxonomic coverage

### Description

**Figure 1.**
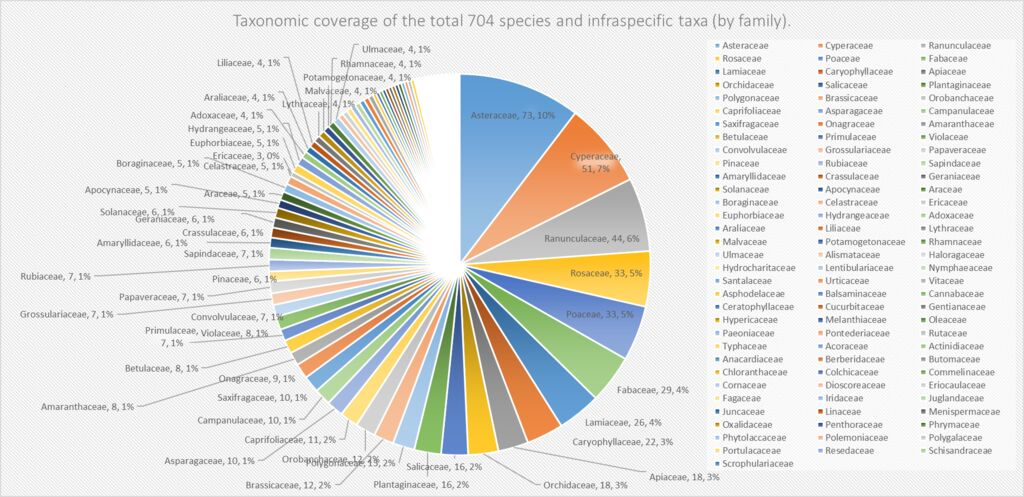
Taxonomic coverage of the first checklist by family: number and percentage of species and infraspecific taxa. Each pie chart represents a family and shows the number and percentage of species and infraspecific taxa within that family. The scientific name of each family is also provided.

This first checklist of spermatophyta and invasive alien plants of Wanda Mountains contains a total of 704 species and infraspecific taxa (six gymnosperm and 698 angiosperm) belonging to 357 genera (four gymnosperm and 353 angiosperm) and 97 families (one gymnosperm and 96 angiosperm) which we represent by family level here (Fig. 1).

### Taxa included

**Table:** 

Rank	Scientific Name	
family	Pinaceae	
family	Nymphaeaceae	
family	Schisandraceae	
family	Chloranthaceae	
family	Acoraceae	
family	Araceae	
family	Alismataceae	
family	Butomaceae	
family	Hydrocharitaceae	
family	Potamogetonaceae	
family	Dioscoreaceae	
family	Melanthiaceae	
family	Colchicaceae	
family	Liliaceae	
family	Orchidaceae	
family	Iridaceae	
family	Asphodelaceae	
family	Amaryllidaceae	
family	Asparagaceae	
family	Commelinaceae	
family	Pontederiaceae	
family	Typhaceae	
family	Eriocaulaceae	
family	Juncaceae	
family	Cyperaceae	
family	Poaceae	
family	Ceratophyllaceae	
family	Papaveraceae	
family	Menispermaceae	
family	Berberidaceae	
family	Ranunculaceae	
family	Paeoniaceae	
family	Grossulariaceae	
family	Saxifragaceae	
family	Crassulaceae	
family	Penthoraceae	
family	Haloragaceae	
family	Vitaceae	
family	Fabaceae	
family	Polygalaceae	
family	Rosaceae	
family	Rhamnaceae	
family	Ulmaceae	
family	Cannabaceae	
family	Urticaceae	
family	Fagaceae	
family	Juglandaceae	
family	Betulaceae	
family	Cucurbitaceae	
family	Celastraceae	
family	Oxalidaceae	
family	Hypericaceae	
family	Violaceae	
family	Salicaceae	
family	Euphorbiaceae	
family	Linaceae	
family	Geraniaceae	
family	Lythraceae	
family	Onagraceae	
family	Anacardiaceae	
family	Sapindaceae	
family	Rutaceae	
family	Malvaceae	
family	Resedaceae	
family	Brassicaceae	
family	Santalaceae	
family	Polygonaceae	
family	Caryophyllaceae	
family	Amaranthaceae	
family	Phytolaccaceae	
family	Portulacaceae	
family	Hydrangeaceae	
family	Cornaceae	
family	Balsaminaceae	
family	Polemoniaceae	
family	Primulaceae	
family	Actinidiaceae	
family	Ericaceae	
family	Rubiaceae	
family	Gentianaceae	
family	Apocynaceae	
family	Boraginaceae	
family	Convolvulaceae	
family	Solanaceae	
family	Oleaceae	
family	Plantaginaceae	
family	Scrophulariaceae	
family	Lentibulariaceae	
family	Lamiaceae	
family	Phrymaceae	
family	Orobanchaceae	
family	Campanulaceae	
family	Asteraceae	
family	Adoxaceae	
family	Caprifoliaceae	
family	Araliaceae	
family	Apiaceae	

## Traits coverage

Overall, the final checklist of spermatophyta and invasive alien plants of the Wanda Mountains contains a total of 704 species and infraspecific taxa, comprised of 656 native plants belonging to 328 genera and 94 families, while 48 invasive alien plants belonging to 39 genera and 20 families were also found. Amongst these, there are 251 new records in native plants and 39 new records in invasive plants. The number of plant species in the Wanda Mountains' area recorded in "Flora Heilongjiangensis" (Zhou 2003) is about 400, while those recorded in the Chinese Virtual Herbarium (CVH 2020) are 227 belonging to 183 genera and 79 families. Therefore, this first checklist is relatively complete.

**Figure 2.**
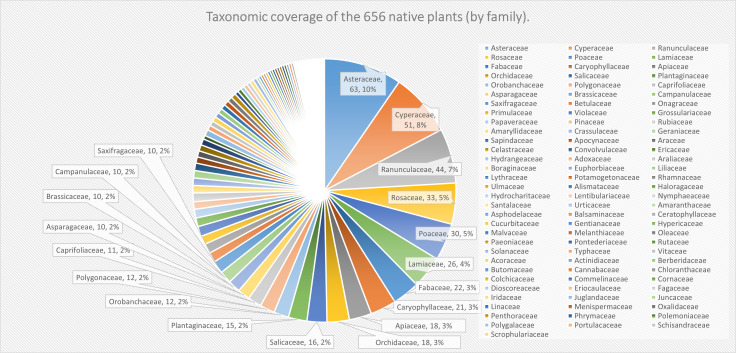
Taxonomic coverage of the 656 native plants by family: number and percentage of species and infraspecific taxa. Each pie chart represents a family and shows the number and percentage of species and infraspecific taxa within that family. The scientific name of each family is also provided.

In total, Asteraceae (63 species and infraspecific taxa), Cyperaceae (51 species and infraspecific taxa) and Asteraceae (10 species), Fabaceae (seven species) emerged as the richest families amongst native and invasive plants, respectively. In regard to native plants, there are eight families with more than 20 species and infraspecific taxa, which are Asteraceae (63), Cyperaceae (51), Ranunculaceae (44), Rosaceae (33), Poaceae (30) Lamiaceae (26) Fabaceae (22) and Caryophyllaceae (21) from the most to the least (Fig. 2). The relatively large families account for 8.51% of the total families and the species that belong to these families account for 44.21% of the total species in this area. In contrast, there are 63 families with five or fewer species and infraspecific taxa and 25 families with only one species and infraspecific taxa. These small families comprise 67.02% of the total families and 21.65% of the total species and infraspecific taxa. This proportion reveals that, on the one hand, dominant families do play an significant role in constituting the main body of the flora of this area and, on the other hand, the Wanda Mountains present a characteristic of complicated species composition with numerous small families.

**Figure 3.**
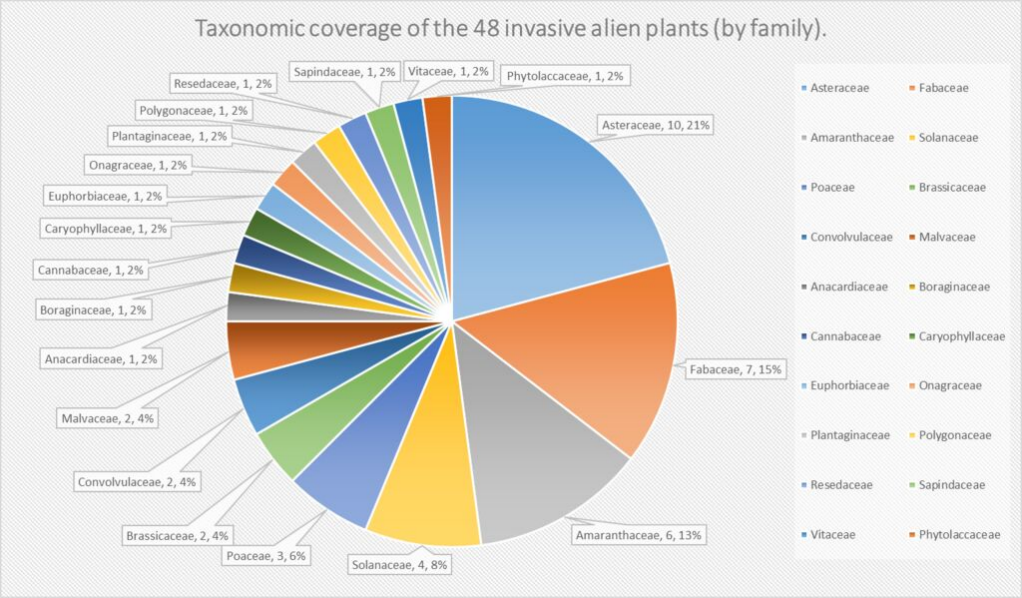
Taxonomic coverage of the 48 invasive alien plants by family: number and percentage of species and infraspecific taxa. Each pie chart represents a family and shows the number and percentage of species and infraspecific taxa within that family. The scientific name of each family is also provided.

**Figure 4.**
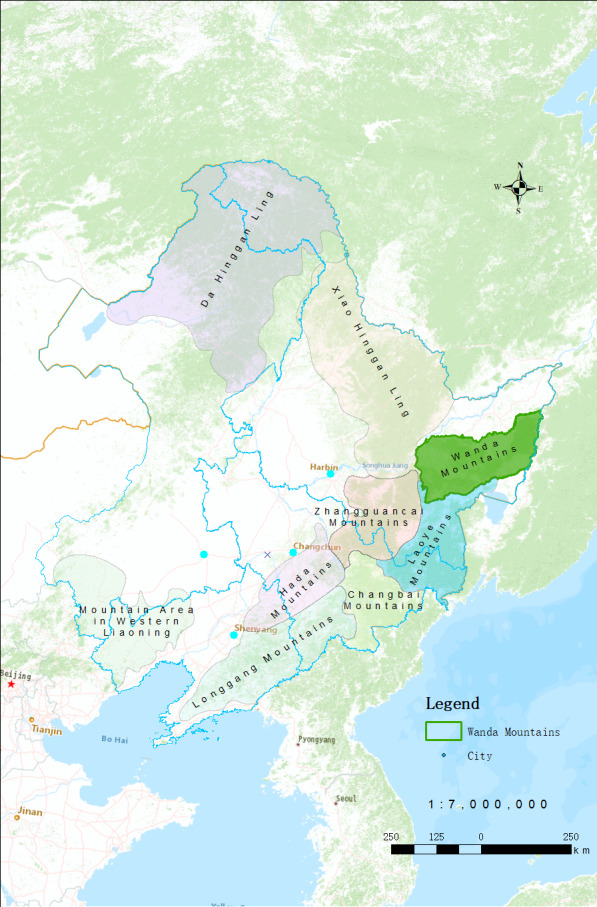
Distribution of mountains in north-eastern China.

**Figure 5.**
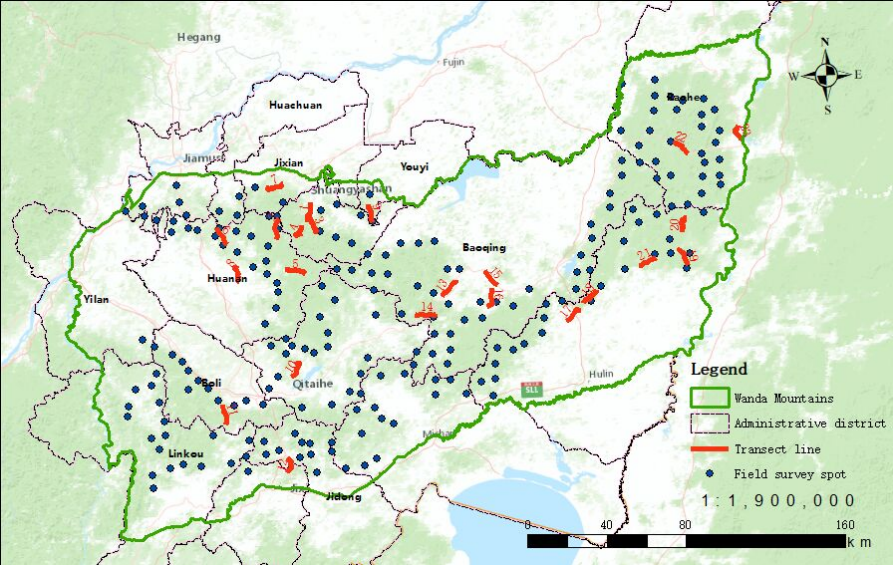
Distribution of the field survey spots and transect lines in Wanda Mountains.

In regard to invasive alien plants, the top three richest families are Asteraceae (10), Fabaceae (7) and Amaranthaceae (6), accounting for 47.92% of 47 species (Fig. 3). In terms of life form, there are 32 annual or biennial herbs (66.67%), 11 perennial herbs (22.92%), four woody plants (8.33%) and one perennial aquatic herb (2.08%) that is *Nasturtiumofficinale*. Some invasive alien plants which have widely invaded Heilongjiang Province, such as *Erigeronannuus*, *Crepistectorum*, *Helianthustuberosus*, *Hibiscustrionum*, *Oenotherabiennis*, *Trifoliumrepens*, *Trifoliumpratense*, *Ambrosiatrifida* and *Seneciovulgaris* occurred on the list as well. During the survey, it is apparent that the invasive alien plants spread along the trafficway strongly presents anthropochory characteristics. According to the survey of invasive plants in Heilongjiang in 2012, the number of invasive plants at that time was 41 species belonging to 35 genera, 17 families (Zheng and Pan 2012), while it has increased to nearly 100 species by 2022 (Wang et al. 2022). In this way, the number shows a trend of rapid growth. Even though we have collected 48 invasive alien plant data, there are some insufficiencies in our survey due to some harmful and widely invasive plants absent from the list, such as *Ambrosiaartemisiifolia*, *Bidensbipinnata* and *Cyclachaenaxanthiifolia*, which are presumed to have invaded the area.

To a certain extent, the first checklist reflects the Spermatophyta profile of the natural ecosystem in Wanda Mountains and gives a stable data basis for the study of flora, biodiversity, vegetation dynamics and preventing the introduction of alien species in this region.

## Temporal coverage

### Notes

The field survey was conducted from June 2018 to September 2020. The data were created from October 2020 until now. Some other field surveys will involve this area so that the data will be updated in the future.

## Usage licence

### Usage licence

Creative Commons Public Domain Waiver (CC-Zero)

## Data resources

### Data package title

Spermatophyta and invasive alien plants of Wanda Mountains in China: a first checklist

### Resource link

Spermatophyta_and_invasive_alien_plants_of_Wanda_Mountains_in_China_a_first_checklist (taibif.tw)

### Number of data sets

1

### Data set 1.

#### Data set name

**firstchecklist_wandamountains_ipt**

#### Download URL

https://ipt.taibif.tw/resource?r=the_first_checklist_of_wanda_mountains1&v=1.9

#### Description

The first checklist of spermatophyta and invasive alien plants of Wanda Mountains contains a total of 704 species and infraspecific taxa, comprising 656 native plants belonging to 328 genera and 94 families, while there are also 48 invasive alien plants belonging to 39 genera and 20 families.

**Data set 1.** 

Column label	Column description
id	id number
taxonID	A unique identifier for the set of nomenclatural and taxonomic information, also shows invasive alien plants with "invasive". For example: "Wanda_2021_1"means one of the 656 native plants; "Wanda_invasive_2021_1" means one of the 48 invasive alien plants.
taxonRank	The taxonomic rank of the most specific name in the scientificName.
ScientificName	The taxon name (with authorship information, if applicable).
institutionCode	Herbarium Code for the PreservedSpecimen records.
catalogNumber	Specimen Code for the PreservedSpecimen records.
decimalLongitude	The geographic longitude (in decimal degrees, using the spatial reference system given in geodeticDatum) of the geographic centre of a Location. Positive values are east of the Greenwich Meridian, negative values are west of it. Legal values lie between -180 and 180, inclusive. We left the value empty if the plant is not a new record.
order	The scientific name of the order in which the taxon is classified.
family	The scientific name of the family in which the taxon is classified.
genus	The scientific name of the genus in which the taxon is classified.
SpecificEpithet	The name of the species epithet of the scientificName.
infraspecificEpithet	The name of the lowest or terminal infraspecific epithet of the scientificName, excluding any rank marker.
scientificNameAuthorship	The authorship information for the scientificName formatted according to the conventions of the applicable nomenclaturalCode.
occurrenceID	A unique identifier for the occurrence. If the plant is only observed in the field, then make the occurrenceID as newrecord-001, newrecord-002..., otherwise recorded-001, recorded-002...
basisOfRecord	The type of the individual record, for example, observation, physical specimen. "HumanObservation" for the new records (field observation only); "PreservedSpecimen" and "MaterialCitation" for the records from physical specimen and published references, respectively.
associatedReferences	Cited publications for MaterialCitation records. We left the value empty if the plant is a new record or recorded by the PreservedSpecimen.
establishmentMeans	The entries "native" and "introduced" are used to present native and invasive plants, respectively.
organismQuantity	A number or enumeration value for the quantity of organisms. Drude's system of abundance 1 to 7 levels are used to describe the quantity from less to more.
organismQuantityType	The type of quantification system used for the quantity of organisms; here we used Drude's system of abundance.
eventDate	The date when the occurrence record was collected, represented as 30/07/2020.
countryCode	A two-letter standard abbreviation for the country of the occurrence locality; in this case, that is CN (China).
geodeticDatum	The ellipsoid, geodetic datum or spatial reference system (SRS), upon which the geographic coordinates given in decimalLatitude and decimalLongitude are based; in this case, that is WGS84.
country	The name of the country in which the Location occurs.
coordinateUncertaintyInMetres	The horizontal distance (in metres) from the given decimalLatitude and decimalLongitude describing the smallest circle containing the whole of the Location. We left the value empty if the plant is not a new record.
decimalLatitude	The geographic latitude (in decimal degrees, using the spatial reference system given in geodeticDatum) of the geographic centre of a Location. Positive values are north of the Equator, negative values are south of it. Legal values lie between -90 and 90, inclusive. We left the value empty if the plant is not a new record.
kingdom	The scientific name of the kingdom in which the taxon is classified.
phylum	The scientific name of the phylum in which the taxon is classified.
class	The scientific name of the class in which the taxon is classified.
